# Photoluminescent F-doped carbon dots prepared by ring-opening reaction for gene delivery and cell imaging†

**DOI:** 10.1039/c7ra13607b

**Published:** 2018-02-07

**Authors:** Tian-Ying Luo, Xi He, Ji Zhang, Ping Chen, Yan-Hong Liu, Hai-Jiao Wang, Xiao-Qi Yu

**Affiliations:** Key Laboratory of Green Chemistry and Technology, Ministry of Education, College of Chemistry, Sichuan University Chengdu 610064 P. R. China jzhang@scu.edu.cn xqyu@scu.edu.cn

## Abstract

Carbon dots (CDs) are photoluminescent nanoparticles with distinctive properties, having great potential in nano-biomaterial systems such as gene/drug delivery vectors and cell imaging agents. Fluorine-doped CD C-6F was prepared by a one-step ring-opening polymerization-dehydrative carbonization (RPDC) approach based on low molecular weight polyethyleneimine (PEI, 600 Da) and fluorinated diglycidyl ethers, while the non-fluorinated counterpart C-6H and the CD prepared from PEI 600 Da solely (C-600) were also prepared for comparison. TEM, FT-IR and XPS were performed to determine the compositions and surface states of the CDs. *In vitro* cell experiment results reveal that the CDs prepared from RPDC approach have much higher transfection efficiency and cellular uptake than PEI 600 contrasts in various cell lines. Compared to non-fluorinated C-6H, C-6F exhibited distinctly higher transfection efficiency, and up to 30 and 260 times higher efficiency than PEI 25 kDa could be achieved in the absence and presence of serum, respectively, indicating the advantage of F-doping. Besides, these CDs exhibit good cell imaging capability under single wavelength excitation, making the materials suitable for cellular tracking and transfection mechanism studies. These results demonstrate that fluorine-doping is an efficient approach to obtained CD gene vectors with high efficiency and serum tolerance.

## Abstract 2

Carbon dots (CDs) are photoluminescent nanoparticles with distinctive properties, having great potential in nano-biomaterial systems such as gene/drug delivery vectors and cell imaging agents.

## Graphical abstract

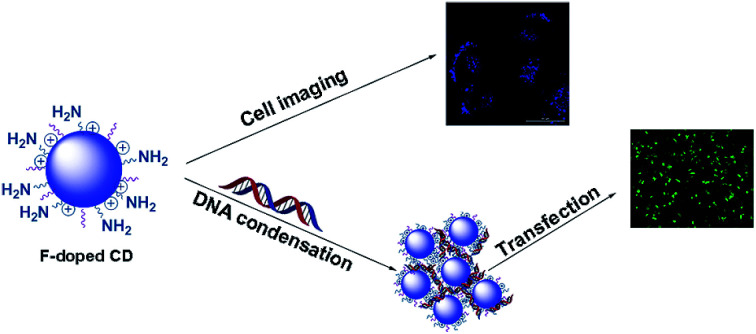

## 1. Introduction

Gene therapy, which can cure various diseases such as acquired diseases and inherited disorders,^1,2^ is considered as a promising therapeutic method in modern medicine. Generally, the efficient delivery of therapeutic genes into targeted cells by proper vector system is vital to the success of gene therapy. Lately, many species of delivery systems have been applied as synthetic vectors for gene delivery (*e.g.* polymers,^3^ dendrimers,^4^ lipids,^5^ gold nanoparticles^6^ and nanodiamonds^7^). Compared to recombinant viral vectors, synthetic non-viral vectors hold several advantages including facile preparation and modification, lack of host immunogenicity, and large-size DNA carrying capacity. However, the medical applications of synthetic vectors are still limited by their potential toxicity and low transfection efficiency (TE).^8–10^ Therefore, it's necessary to design and develop excellent non-viral gene vectors with high TE and biocompatibility.

As a new type of fluorescent nanomaterial, carbon dots (CDs) have attracted intensive attentions over the past decade because of their numerous merits including facile preparation and functionalization, good photostability, excellent water dispersibility and biocompatibility.^11,12^ Thus, CDs have been made impressive progress in many promising applications such as cell imaging, bio-sensing, theranostic and light-emitting devices.^13–16^ In addition to traditional CDs, heteroatom doping into the CD has become a powerful strategy to obtain advanced quantum dot materials. To date, various heteroatoms, such as N,^17^ S,^18^ P,^19^ B,^20^ Mg,^21^ Ge and Si,^22,23^ have been doped into CDs, and these studies showed that heteroatom doping could improve the fluorescence property together with biocompatibility, cellular uptake, and stability.

In recent years, CDs were employed for theranostic applications such as simultaneous gene delivery and intracellular tracking. Their good performance attracted extensive interests from researchers in relative fields. Wu and co-workers used bPEI as carbon source to construct CDs by a hydrothermal approach, and these CD showed extremely low cytotoxicity and good efficiency for *in vitro* transfection.^24^ Pierrat *et al.* reported a microwave irradiation method to synthesize CDs as efficient gene carriers with citric acid and bPEI 25 kDa as precursors.^25^ More recently, our group also proved that introduction of hydrophobic tails on CD might lead to much enhanced gene transfection efficiency in human A549 cells with some “self-targeting ability”.^12^ Inspired by these results, we hoped that heteroatom doping may further improve the gene delivery performance of the CD materials.

**Scheme 1:**
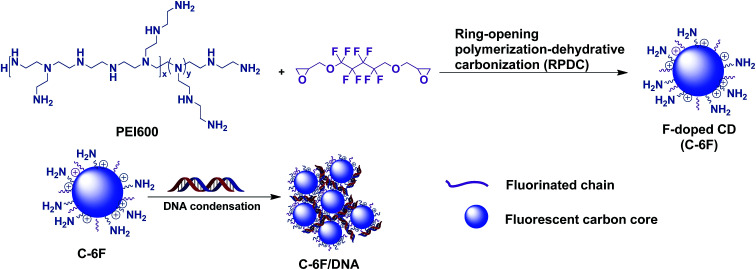
Schematic illustration of the formation of F-doped CDs and their interaction with DNA.

Considering the advantages brought by fluorinated moieties for non-viral gene vectors such as cationic polymers and dendrimers,^26,27^ Herein, we first report the successful preparation of a new-type F-doped CD *via* a novel ring-opening polymerization-dehydrative carbonization (RPDC) method by using PEI 600 Da and fluorinated diglycidyl ethers as precursors (Scheme 1). The F-doped CDs were used for cell imaging and gene delivery, results reveal that the F-doped CD has low cell toxicity and high transfection efficiency with excellent serum tolerance. Meanwhile, it was found that such CD material could act as good cell imaging agent under single wavelength excitation, making the materials suitable for cellular tracking and transfection mechanism study.

## 2. Materials and methods

### 2.1. Reagents and chemicals

All chemicals and reagents were obtained commercially and were used as received. The ^1^H NMR spectra and ^19^F NMR spectra were measured on a Bruker AM400 NMR spectrometer. Fourier transform infrared (FT-IR) spectra was performed on a FT-IR Nicolet 380 spectrometer. TEM observations were performed on TecnaiG2 F20 S-TWIN. X-ray photoelectron spectra (XPS) of the CDs was performed on an Axis Ultra DLD spectrograph with Al/Kα as the source. The composition of C-6H and C-6F was determined by elemental analysis on an Elementar Vario EL III apparatus. Luciferase assay kit was purchased from Promega (Madison, WI, USA). Endotoxin-free plasmid purification kit was purchased from TIANGEN (Beijing, China). PEI600 (branched, average *M*_w_ = 600 Da) was purchased from Aladdin (Shanghai, China). PEI25K (branched, average *M*_w_ = 25 kDa) was purchased from Sigma-Aldrich (St Louis, MO, USA). The pUC19 DNA was purchased from Thermo Scientific. The pGL-3 (Promega, Madison, WI, USA) coding for luciferase DNA as the plasmids were used in the study. Cy5 was purchased from Mirus Bio, LLC (Madison, WI). The Dulbecco's modified Eagle's medium (DMEM), 1640 Medium and fetal bovine serum (FBS) were purchased from Invitrogen Corporation. Cellular uptake of plasmid DNA (Flow Cytometry) was carried out following previously reported procedures.^27^ HepG2 (human hepatocellular carcinoma cell line), HeLa (human cervical carcinoma cell line), 7702 (normal human liver cell line) and A549 (human lung carcinoma cell line) were purchased from the Shanghai Institute of Biochemistry and Cell Biology, Chinese Academy of Sciences.

### 2.2. Preparation of CDs

#### C-600

PEI 600 Da (0.4 g) was dissolved in anhydrous ethanol (10 mL), and the solution was then transferred to a poly(tetrafluoroethylene) (Teflon)-lined autoclave (100 mL) and heated at 180 °C for 12 h. After heating, the reactor was cooled to room temperature. The obtained brown solution was filtered by a 450 nm film filter, and then dialyzed (M. wt = 3500 Da) against deionized water for 24 h. The product was obtained by lyophilization.

#### C-6H and C-6F

These materials were prepared through the same procedures as that for C-600, except using additional 1,5-pentylene glycol diglycidyl ether (0.2 g, for C-6H) or 2,2,3,3,4,4-hexafluoro-1,5-pentanediol diglycidyl ether (0.2 g, for C-6F).

### 2.3. Agarose gel retardation assay

Material/DNA complexes at different mass ratios ranging from 0.25 to 8.0 (mass ratio of material relative to DNA) were prepared by adding an appropriate volume of material to pUC-19 DNA (0.125 mg). The complexes solution obtained was diluted to a total volume of 10 μL and then incubated at 37 °C for 30 min, 2.5 μL loading buffer was added and then solution was electrophoresed on the 1% (WV-1) agarose gel containing GelRed at 120 V for 40 min. The nucleic acid was visualized with a UV lamp using a BioRad Universal Hood II.

### 2.4. Dynamic light scattering measurements

The average particle size and zeta potential of complexes were measured by DLS using a Nano-ZS 3600 (Malvern Instruments, USA) with a He–Ne Laser beam (633 nm, fixed scattering angle of 90°) at 25 °C. Complexes at different mass ratios were prepared by adding 1 μg of pUC-19 DNA to the appropriate volume of the material solution (in ultrapure water). Then the solution of the complexes was incubated at 37 °C for 30 min and then diluted with deionized water to 1 mL prior to measurement.

### 2.5. Quantum yields measurements

The QY of CDs was analysed by a comparative optical method. Quinine sulfate aqueous (quantum yield = 54%) was selected as a standard sample. Absolute quantum yield was calculated according to the following equation:QY = QY_s_(*F*_CDs_/*F*_s_)(*A*_s_/*A*_CDs_)where QY was the quantum yield, *F* was the measured integrated emission intensity, and *A* was optical density. The subscript “s” refers to standard with known quantum yield and “CDs” for the test sample. In order to minimize fluorescence quenching, absorbance in the 10 mm fluorescence cuvette was kept below 0.10 at the excitation wavelength (360 nm).

### 2.6. Cell culture

HepG2 cells and HeLa cells were incubated in DMEM containing 10% FBS and 1% antibiotics (penicillin–streptomycin, 10 kU mL^−1^), while 7702 cells and A549 cells were incubated in 1640 medium containing 10% FBS and 1% antibiotics (penicillin–streptomycin, 10 kU mL^−1^) at 37 °C in a humidified atmosphere containing 5% CO_2_.

### 2.7. Cell viability assay

To evaluate biocompatibility of material, samples were carried out with 7702 and HeLa cells by CellTiler 96® AQ_ueous_ One Solution Cell Proliferation Assay. Briefly, about 1 × 10^5^ cells per well were seeded in 96-well plates and cultured 24 h. After 24 h, PEI600, C-600, C-6H and C-6F were complexed with 0.2 μg of DNA at various weight ratios for 30 min. Then the medium was exchanged with a serum containing culture medium (100 μL) containing the complexes. The cells were further incubated for 24 h. After that, the solutions were removed, 100 μL PBS contained 20 μL CellTiler 96® AQ_ueous_ One Solution Cell Proliferation was added to each well for additional 1 h incubation at 37 °C. Then, the absorbance of each sample was measured using an ELISA plate reader (model 680, BioRad) at a wavelength of 490 nm. The cell survival was expressed as follows: cell viability = (OD_treated_/OD_control_) × 100%. The untreated cell controls were taken as 100% cell viability. The cell viability of 25 kDa PEI was also performed.

### 2.8. Gene transfection procedure

Gene transfection of a series of complexes was investigated in 7702 cells (6.0 × 10^4^ cells per well), HeLa cells (6.0 × 10^4^ cells per well), A549 cells (6.0 × 10^4^ cells per well), HepG2 cells (6.0 × 10^4^ cells per well), which were seeded in 48-well plates and grown to reach 70–80% cell confluence at 37 °C for 24 h in 5% CO_2_. Before transfection, the medium was replaced serum free or a serum containing culture medium containing material/pDNA (0.4 μg) complexes at various mass ratios. After 4 h under standard incubator conditions, the medium was replaced with fresh medium containing serum and incubated for another 20 h.

For fluorescent microscopy assays, cells were transfected by complexes containing pEGFP-N1. After 24 h incubation, EGFP-expressed cells were observed with an inverted fluorescence microscope (Nikon Eclipse TE 2000E) equipped with a cold Nikon camera.

The luciferase assay was performed according to the manufacturer's protocols (Promega). For a typical assay in a 48-well plate, 24 h post transfection as described above, cells were rinsed twice with PBS and lysed with 60 μL 1× Lysis reporter buffer (Promega). The total protein was measured using a BCA protein assay kit (Pierce). Luciferase activity was expressed as relative light units (RLU) per mg protein. All the experiments were done in triplicates.

### 2.9. Cellular uptake of plasmid DNA

The cellular uptake of the material/Cy5-labeled DNA complexes was determined using flow cytometry. The Label IT Cy5 Labeling Kit was used to label pDNA according to the manufacturer's protocol. Briefly, 7702 cells (1.2 × 10^5^ cells per well) and HeLa cells (1.2 × 10^5^ cells per well) were separately seeded in 24-well plates, and allowed to attach and grow for 24 h. Cells were incubated with complexes containing Cy5-labeled DNA (0.8 μg of DNA per well, optimal mass ratio of each sample) in media for 4 h at 37 °C. Subsequently, the cells were washed once with PBS and harvested with 0.25% trypsin/EDTA and resuspended in PBS. Mean fluorescence intensity was analyzed using flow cytometer (Becton Dickinson and Company). Cy5-Labeled plasmid DNA uptake was measured in the FL4 channel using the red diode laser (633 nm).

### 2.10. Confocal laser scanning microscopy (CLSM) analysis

7702 cells or HeLa cells were seeded in a 35 mm confocal dish (*Φ* = 15 mm) at a density of 2 × 10^4^ cells per well, and the cells were allowed to attach and grow for 24 h. The medium was exchanged with serum-containing medium. For cell imaging, cells were incubated with CDs (10 μg mL^−1^) for 4 h at 37 °C.

## 3. Results and discussion

### 3.1. Preparation and characterization of the CDs

**Fig. 1:**
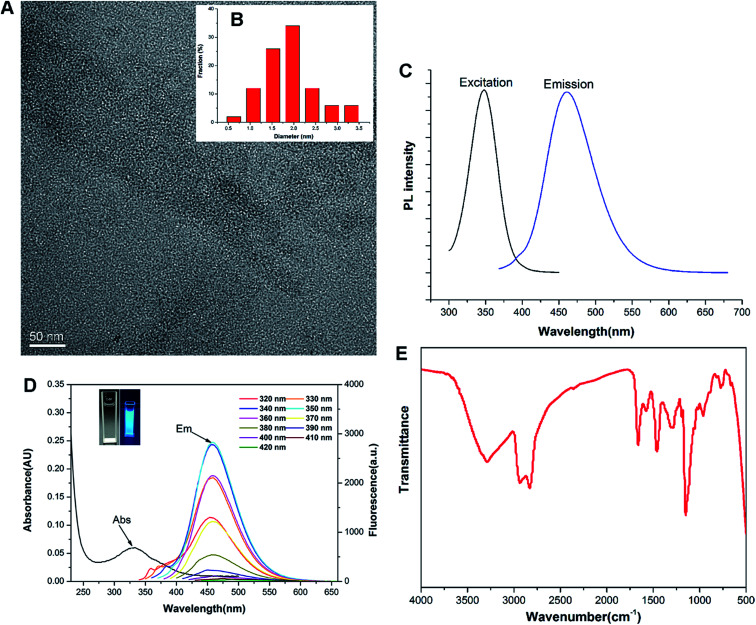
Characterization of C-6F. (A) TEM image; (B) size distribution histogram from TEM; (C) luminescence excitation (black line) and emission spectra (blue line); (D) absorption curves and PL emission spectra under different *λ*_ex_ (inset: C-6F aqueous solution under daylight and UV light); (E) FT-IR spectrum.

Previous studies revealed that the polyamine compounds could be polymerized smoothly by using diglycidyl ethers as linking groups.^28,29^ Accordingly, the F-doped CD can be facilely synthesized through RPDC approach (Scheme 1). In such method, the water-dispersible F-doped CD (C-6F) was prepared solvothermally by treating PEI 600 Da and fluorinated diglycidyl ethers in anhydrous ethanol solution followed by dialysis purification. Meanwhile, the non-fluorinated counterpart C-6H and the CD prepared from PEI 600 Da solely (C-600) were also prepared for comparison. ^1^H and ^19^F NMR spectra (Fig. S1 and S2†) revealed the existence of PEI and CF_2_ structures. Subsequent TEM study exhibited that the particle size of C-6F was uniformly distributed in the range of 1.5–3.5 nm (Fig. 1A and B), and this was similar to the results of C-6H (Fig. S3†). Elemental analysis (Table S1†) showed that by comparison to C-6H, the fluorinated CD gave a decrease of H content and slight decrease of N and C contents, also suggesting the successful conjugation of the fluorines. The photoluminescent properties of C-6F were examined, the maximum excitation wavelength (*λ*_ex_) and maximum emission wavelength (*λ*_em_) was found as to be 348 and 460 nm, respectively (Fig. 1C). The full width at half maximum (FWHM) at excitation of 348 nm was only 75 nm, which was smaller than previously reports.^24,30^Fig. 1D shows the UV/Vis absorption spectra of C-6F with local absorption at 330 nm. The aqueous solution of C-6F showed a significant blue fluorescence when irradiated by UV light. In addition, photoluminescence spectra of C-6F at *λ*_ex_ from 320 to 420 nm did not give apparent shift of *λ*_em_, which was different from some other reported CDs.^12,31^ The counterpart C-6H also gave similar results (Fig. S4 and S5†). Quinine sulfate in 0.10 M of H_2_SO_4_ was selected as a standard sample to calculate the quantum yields (QY) of CDs, and the QYs of C-6H and F-doped C-6F were found to be 6.6% and 5.6%, respectively (Table S2†).

**Fig. 2:**
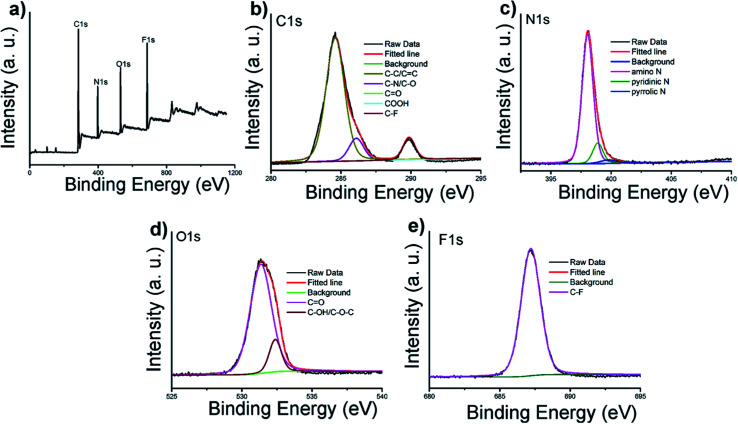
(a) XPS survey of C-6F; (b–e) high resolution XPS spectra of C 1s, N 1s, O 1s, and F 1s.

FT-IR and XPS were also performed to determine the compositions and surface states of C-6F. As shown in Fig. 1E (and Fig. S6† for C-6H), the broad bands centered at 3288 cm^−1^ are attributed to O–H and N–H stretching vibrations, suggesting the existence of hydroxyl and amino groups on the surface. The peaks between 2829 and 2936 cm^−1^ are attributed to C–H stretching vibrations of methyl/methylene, while the peaks at 1662, 1577, 1460 and 1287 cm^−1^ are attributed to C=N/C=O, N–H, CH_2_ and C–N bonds respectively.^11,31^ The peak at 1150 cm^−1^ can be ascribed to C–O stretching vibrations. The XPS surveys supported these FT-IR analyses. The full spectra presented in Fig. 2a show four typical peaks at 284, 397, 529 and 686 eV, which are attributed to C 1s, N 1s, O 1s, and F 1s, respectively, indicating C-6F mainly containing these four elements (and Fig. S7† for C-6H).^17,32^ High resolution XPS spectrum of C 1s (Fig. 2b) fitted with five peaks at 284.6, 286.1, 287.8, 288.8 and 289.9 eV, which were attributed to the C–C/C=C, C–N/C–O, C=O, COOH and C–F bonds, respectively.^33,34^ For N 1s, three peaks at 398.1, 398.9 and 399.8 eV could be assigned to amino N, pyridinic N and pyrrolic N, respectively (Fig. 2c).^11,32,35,36^ The O 1s band could be deconvoluted into two binding energies at 531.4 and 532.4 eV, representing C=O and C–OH/C–O–C, respectively (Fig. 2d).^13^ The XPS spectrum of F 1s was resolved into one peak at 687.2 eV, which was attributed to C–F bond (Fig. 2e).^34^ Above results suggested the formation of aromatic core structure together with functional groups on the surface of the prepared CD.

### 3.2. Interaction with DNA

**Fig. 3:**
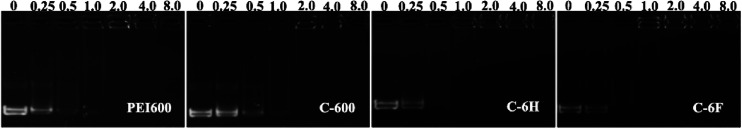
Agarose gel electrophoresis of complexes at different mass ratios, w/w of 0 refers to DNA control.

The ability to condense DNA into nanosized particles is a crucial factor for efficient cell transfection. Gel retardation assay was performed to confirm the interaction between DNA binding ability between the CDs and plasmid DNA at various mass ratios (w/w), and the images are shown in Fig. 3. With the increase of mass ratio, the intensity of DNA migration bands in the agarose gel decreased. All the CDs exhibited good DNA retardation ability, and the total retardation could be achieved below the w/w of 4, compared to C-600, C-6H and C-6F gave slightly higher binding ability, and they could completely inhibit the DNA migration from w/w of 1. Such results suggest that the CDs prepared from RPDC method have higher surface positive charge density, resulting in easier binding toward negatively charged DNA through electrostatic interaction.

**Fig. 4:**
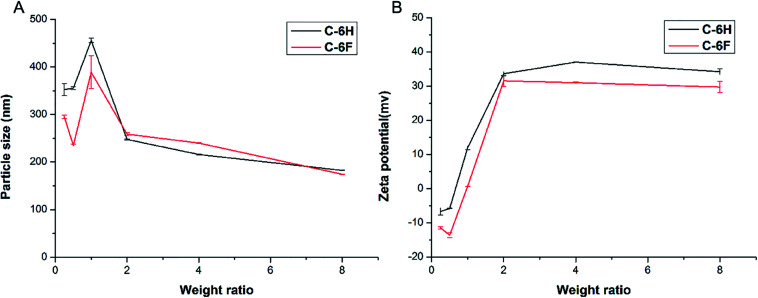
Average particle size (A) and zeta-potential (B) of CDs/DNA complexes obtained at various mass ratios using DLS (data represent mean ± SD, *n* = 3).

Appropriate size and zeta potential of complexes are of critical importance for the gene carriers, these factors can even affect the endocytosis pathway of the nanoparticles.^37^ DLS was performed to characterize these properties of the CDs/DNA complexes at different mass ratios (w/w = 0.25, 0.5, 1.0, 2.0, 4.0, 8.0). As shown in Fig. 4, the particle size of the complexes tended to be steady with the increase of mass ratio, and finally reached the size of around 200 nm. It was reported that complexes within the size range of 50 to several hundred nanometers were suitable for cell uptake.^28,38,39^ On the other hand, with the increase of w/w, the positive charge also reached a plateau of +30 to 40 mV for both complexes. In general, nanoparticles with higher positive charge exhibited a stronger affinity for the negatively charged cell membrane, resulting in higher cellular uptake.^40,41^ Besides, the morphology of the C-6F/DNA complexes was further investigated by TEM. The F-doped CD in deionized water could condense DNA into spherical nanoparticles with a diameter of 20–30 nm (Fig. S8†). Compared with the results obtained from DLS, the particle sizes measured by TEM were smaller. The reason might be that the particle sizes measured obtained by TEM had been dried after being dropped onto carbon-coated copper meshes, while those measured by DLS were in the hydrated state in solution.^29^

### 3.3. Cytotoxicity

**Fig. 5:**
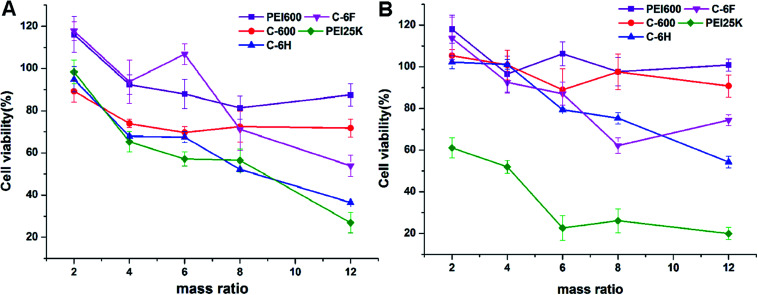
Cytotoxicity of the CD/DNA complexes at different mass ratios toward 7702 (A) and HeLa (B) cells. PEI/DNA polyplexes were used for comparison. Data represent mean ± SD (*n* = 3).

High cytotoxicity is always one of the major barriers that severely limits the use of biomaterials *in vivo* and clinical applications. The cell viability after 24 h incubation with PEI 600 Da, C-600, C-6H, C-6F and PEI 25 kDa was evaluated in 7702 and HeLa cells by using a standard MTS cell viability assays. Results in Fig. 5 show that the toxicity trends in two cell lines are similar. As expected, the cytotoxicity of CDs were much lower than high molecular weight PEI, and were comparable to the lower molecular weight PEI species. Previous reports have found that fluorinated biomaterials were low cytotoxic and could be used as supported scaffolds for cell culture and tissue engineering.^42,43^ The C-6F/DNA complexes showed minimal cytotoxicity on the 7702 cells (Fig. 5A). And only slight decreases in cell viability (10%) were observed for the C-6F/DNA complexes on HeLa cells (Fig. 5B). Besides, the C-6F complex also show lower cytotoxicity in 7702 cells than C-6H contrast.

### 3.4. *In vitro* gene transfection

**Fig. 6:**
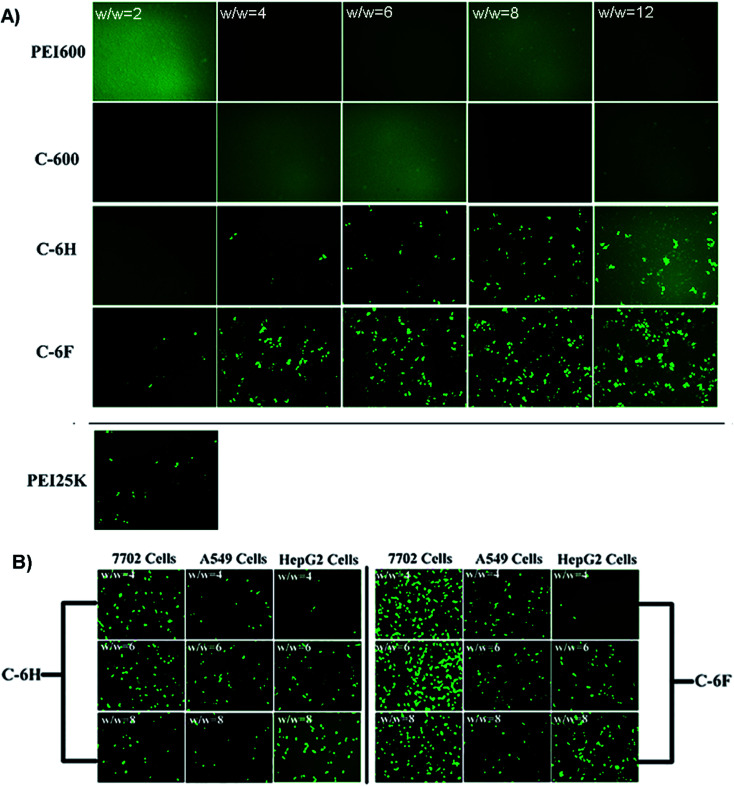
(A) Fluorescence microscope images of HeLa cells transfected by various vectors. (B) C-6H and C-6F mediated EGFP expression in other cell lines (7702, A549 and HepG2 cells).

To initially evaluate the gene delivery capability of the CDs, direct visualization of gene expression of enhanced green fluorescent protein (EGFP) was observed by an inverted fluorescent microscope. As shown in Fig. 6A, neither PEI 600 Da nor C-600 could mediate any EGFP expression in HeLa cells. On the contrary, the CDs prepared through RPDC approach (C-6H and C-6F) gave much better transfection, especially at relatively higher w/w ratios. Between these two CDs, the fluorinated C-6F induced notably stronger fluorescence signals, indicating the advantage of F-doping, and the TE was also much higher than the “golden standard” PEI 25 kDa. Moreover, transfection experiments were also performed in several other cell lines (7702, A549 and HepG2 cells, Fig. 6B). These two CD mediated moderate EGFP expression in A549 and HepG2 cells. However, in 7702 cells, C-6F could induce significantly stronger EGFP fluorescence at relatively lower w/w ratios (4 and 6). These results also suggest that the F-doping approach is an effective strategy to enhance the gene transfection ability of CD materials.

**Fig. 7:**
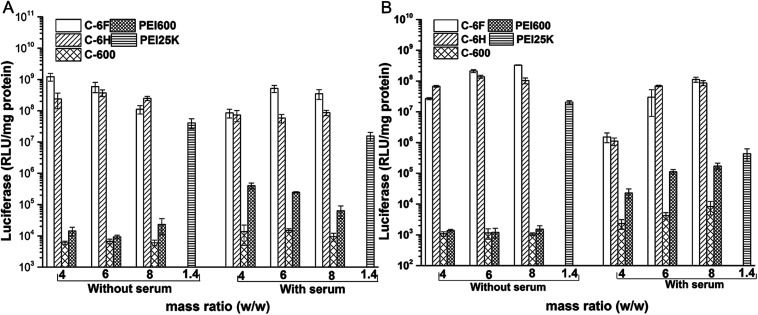
Luciferase gene expression in 7702 (A) and HeLa (B) cells transfected by complexes at different mass ratios in comparison with 25 kDa PEI (w/w = 1.4).

The transfection by the materials were further investigated quantitatively in 7702 and HeLa cells by luciferase transfection assay. Experiments were performed in both serum-free and serum-containing conditions with the use of PEI 25 kDa at its optimal w/w ratio (1.4) for comparison, and the results are shown in Fig. 7. It was revealed that in most case, C-600 and PEI 600 gave poor TE, while the CDs prepared through RPDC approach (C-6H and C-6F) could induce much better transfection, and the TE were also higher than PEI 25 kDa. Under optimized condition, C-6F could give 30 and 16 times higher TE than PEI in 7702 and HeLa cells, respectively, and the optimized TEs were also higher than non-fluorinated counterpart C-6H. More importantly, in the serum circumstance, the TE of PEI was largely suppressed, however, the transfection by the CDs did not be inhibited obviously, indicating their good serum tolerance. For example, with the presence of serum, C-6F gave comparable TE to that obtained without serum in 7702 cells. In Hela cells, up to 260 times higher TE than PEI could be achieved in the presence of serum. To further confirm the preferable serum tolerance of the RPDC CDs, C-6F and C-6H were chosen to study their transfection behavior under different serum concentrations (0, 10, 20, 30 and 50%) in these cell lines. Unlike those of PEI and C-6H, the TE of C-6F was better maintained with the addition of serum, and the relative TE (compared to those of PEI and C-6H) increased with the rise of serum concentration (Fig. S9†). Such advantage of C-6F might be attributed to its special structure, which contained both hyperbranched polyamine and doped fluoroalkyl chain.^26,29^

**Fig. 8:**
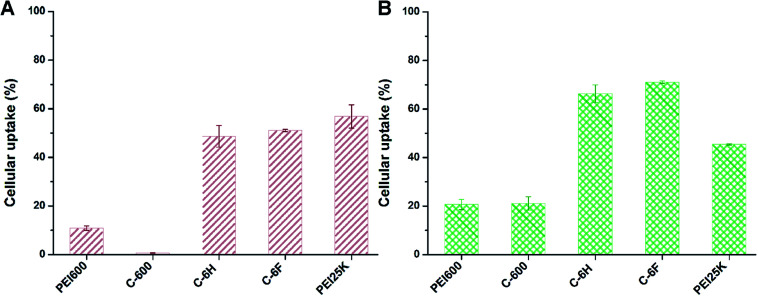
Cellular uptake of complexes at optimal mass ratios in 7702 cells (A) and HeLa cells (B) quantified by flow cytometry analysis. Data represent mean ± SD (*n* = 3).

As one of the main barriers in the gene delivery process, the internalization of the nucleic acid complexes largely influences the TE.^44^ Flow cytometry was applied to analyze the cellular uptake of the complexes after incubation of 7702 and HeLa cells with complexes containing Cy5-labeled DNA. In 10% serum-containing condition, the incubation was processed at the optimized mass ratio for 4 h. The percentages of Cy5-positive cells was calculated. As shown in Fig. 8, compared to PEI 600 and its CD derivative C-600, the CDs prepared from RPDC method (C-6H and C-6F) gave much higher cellular uptake, especially in HeLa cells. This might contribute much to their higher TE, and also proved that RPDC approach is a promising strategy for the improvement of gene transfection performance of CDs.

### 3.5. Cell imaging property of the CDs

**Fig. 9:**
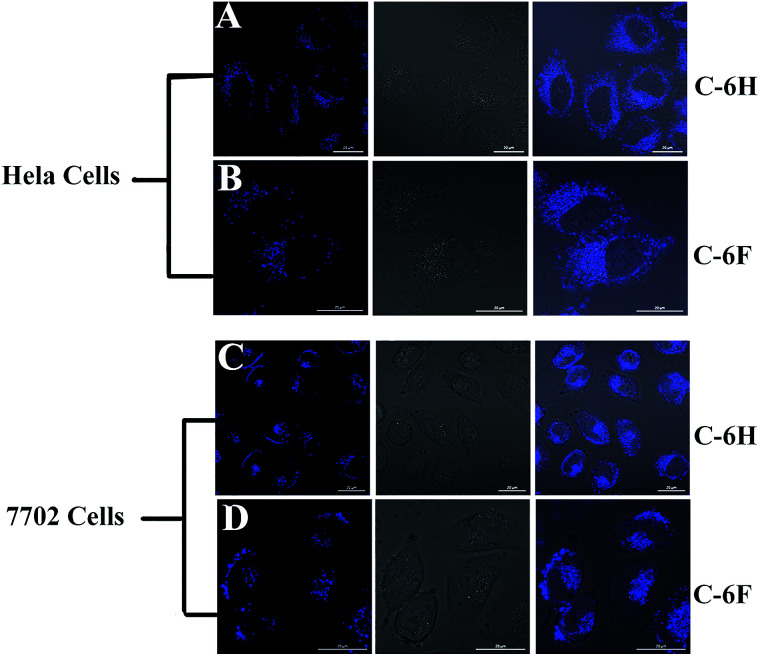
Confocal fluorescence images of HeLa and 7702 cells incubated with 10 μg mL^−1^ of C-6H and C-6F. (A and C) For C-6H, (B and D) for C-6F, for each row from left to right: excited at 405 nm, bright field, merged image. Scale bar = 20 μm.

Since CDs have high PL stability and good biocompatibility, the as-prepared CDs were expected to potentially serve as cell imaging agents. The cell samples were incubated for 4 h with the C-6F and C-6H (10 μg mL^−1^) before imaging by confocal laser scanning microscopy (CLSM). As shown in Fig. 9, after being incubated with the CDs, both 7702 and HeLa cell lines emitted strong blue fluorescence under illumination at a single wavelength of 405 nm. The fluorescence emissions predominantly located in the cytoplasm, indicating that the two CDs could smoothly pass cell membrane and enter the cells.

## 4. Conclusion

In summary, we developed a polymerization-dehydrative carbonization (RPDC) method to prepare photoluminescent F-doped CDs. These CDs (C-6F and its non-fluorinated counterpart C-6H) could well condense DNA into nanoparticles with proper size and surface charge. Compared to the CD formed from solely PEI 600, they could give much higher transfection efficiencies and cellular uptake. Between the CDs prepared *via* RPDC method, the fluorinated C-6F exhibited distinctly higher transfection efficiency than C-6H, indicating the advantage of F-doping, and the efficiency was also much higher than the “golden standard” PEI 25 kDa. Further, serum has little negative effect on the transfection by C-6F. Compared to C-6H and PEI, its transfection efficiency was seldom reduced even with high serum concentration. On the other hand, these CDs exhibited good cell imaging capability under single wavelength excitation, making the materials suitable for cellular tracking and transfection mechanism study. These results suggest that F-doping approach can effectively improve the gene transfection efficacy of CDs. Further optimization studies and extension of their application are now in progress.

## Conflicts of interest

There are no conflicts to declare.

## Supplementary Material

RA-008-C7RA13607B-s001
