# Supplementary material for: Photoluminescent F-doped carbon dots prepared by ring-opening reaction for gene delivery and cell imaging
Source: RSC Adv. 2018 Feb 7;8(11):6053–62. doi: 10.1039/c7ra13607b (PMC9078219; doi:10.1039/c7ra13607b)
Supplement: RA-008-C7RA13607B-s001 [file RA-008-C7RA13607B-s001.pdf]

Electronic Supplementary Material (ESI) for RSC Advances.
This journal is © The Royal Society of Chemistry 2018

Supplementary Information

Photoluminescent F-doped Carbon Dots Prepared by Ring-Opening Reaction for Gene Delivery and Cell Imaging

Tian-Ying Luo, Xi He, Ji Zhang*, Ping Chen, Yan-Hong Liu, Hai-Jiao Wang and Xiao-Qi Yu*

Key Laboratory of Green Chemistry and Technology (Ministry of Education), College of Chemistry, Sichuan University, Chengdu 610064, P. R. China

*Corresponding authors: jzhang@scu.edu.cn (J. Zhang); xqyu@scu.edu.cn (X.-Q. Yu).

Tables and figures

Table 1. The C, N and H of content of CDs by elemental analysis

	N(%)	C(%)	H(%)
**C-6H**	14.4	55.9	11.9
**C-6F**	13.8	46.4	7.6

Table 2. Quantum yield (QY) of CDs using quinine sulfate as a reference.

	F_{CDs}	A_{CDs}	QY
**C-6H**	244722.9395	0.044	6.6%
**C-6F**	186586.6375	0.040	5.6%

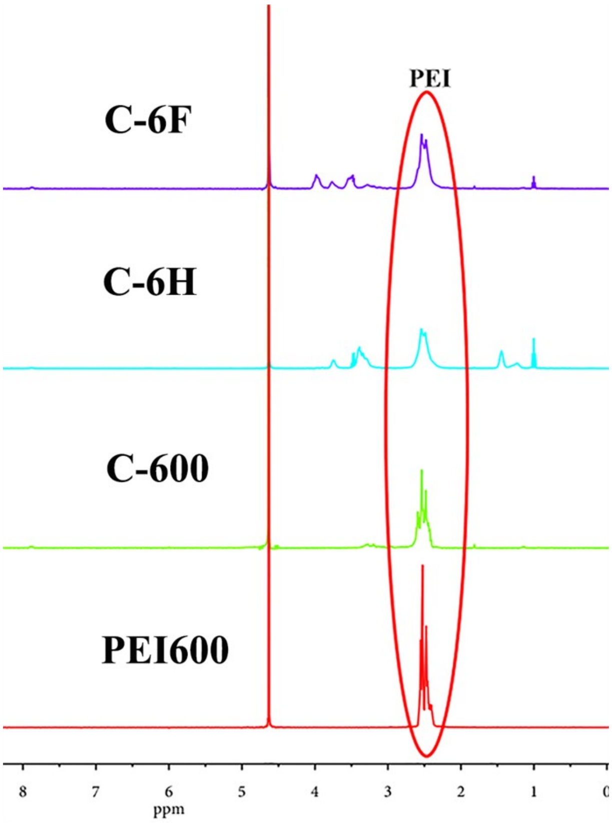

Fig. S1. ^{1}H NMR spectra of the CDs

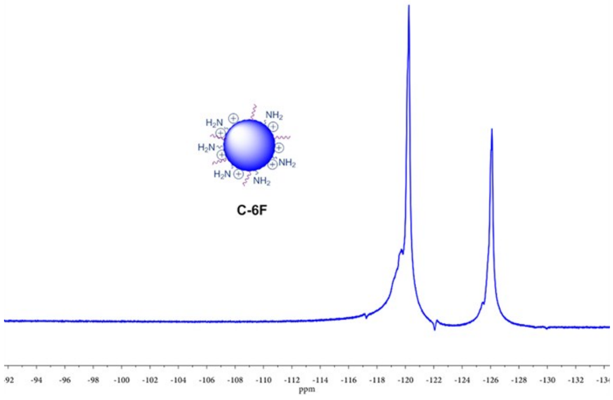

Fig. S2. ^{19}F NMR spectrum of **C-6F**.

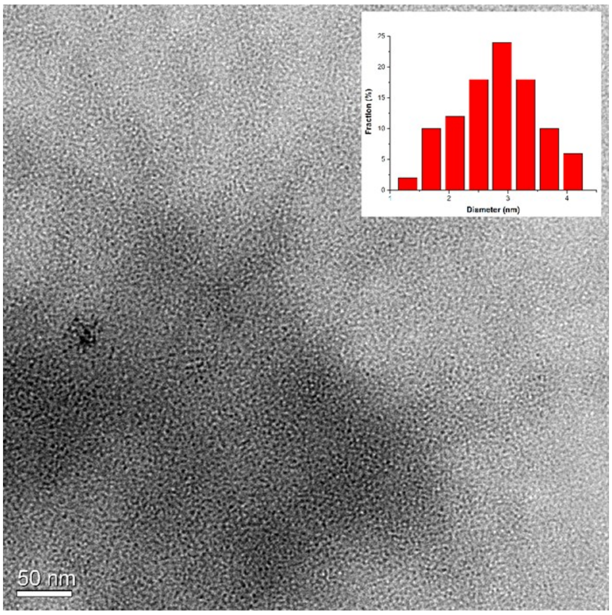

Fig. S3. TEM image of **C-6H**.

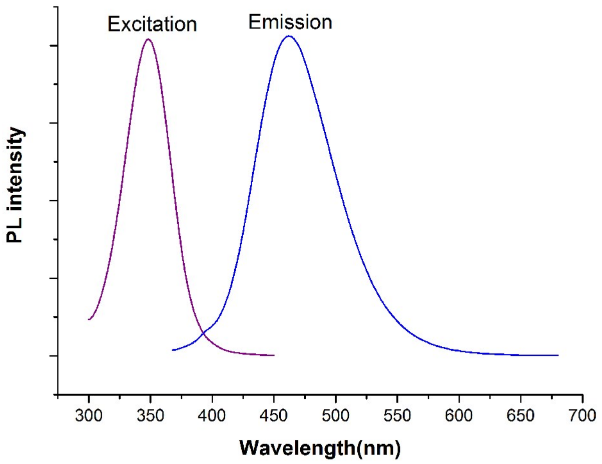

Fig. S4. Luminescence excitation (purple) and emission spectra (blue) of **C-6H**

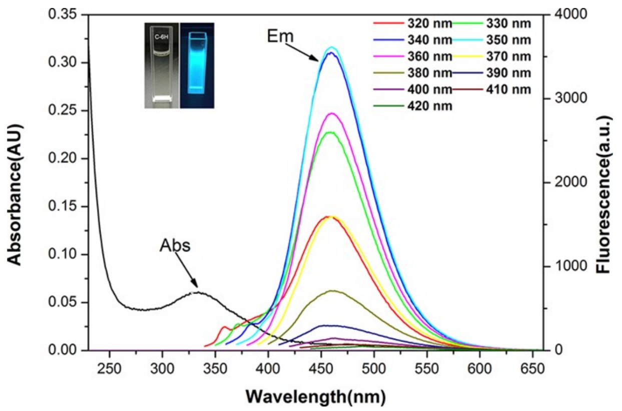

Fig. S5. Absorption curves (Abs) and PL emission spectra (Em) under excitation with light of different wavelengths (**C-6H** aqueous solution under daylight (left) and UV light in the inset.)

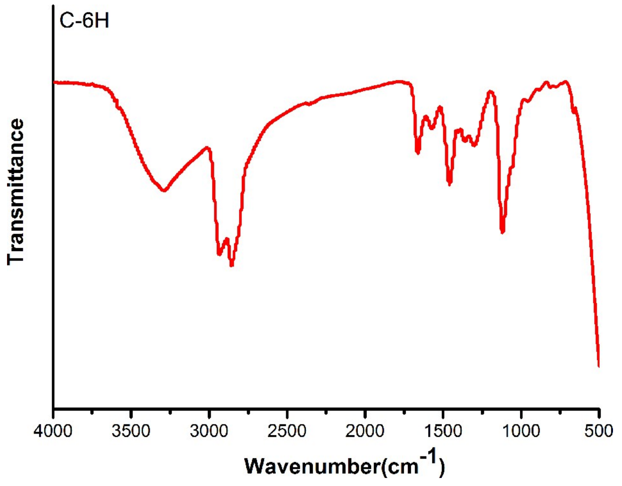

Fig. S6. FT-IR spectrum of **C-6H**.

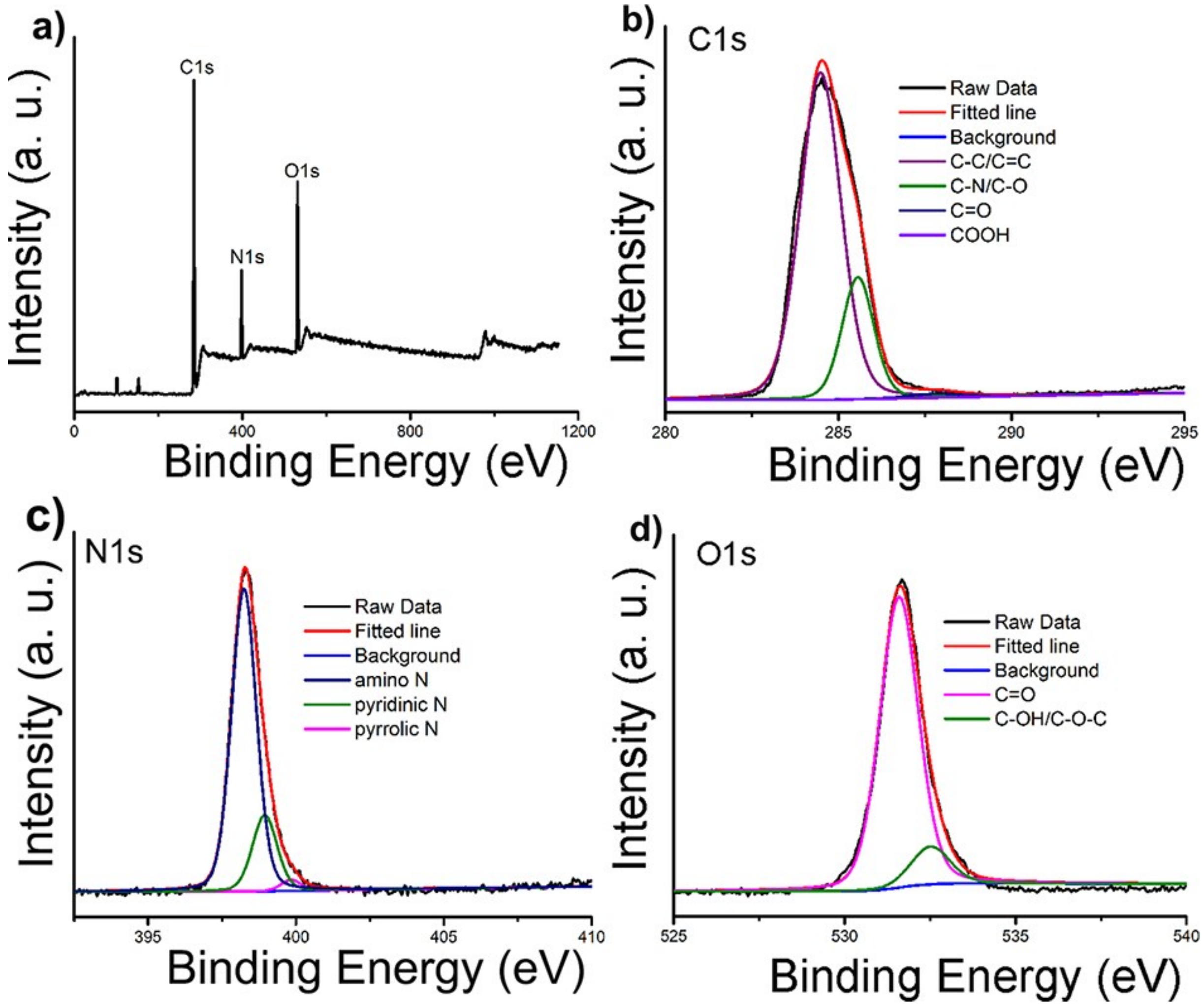

Fig. S7. (a) XPS survey; and (b–e) high resolution XPS spectra of C 1s, N 1s, O 1s of **C-6H**.

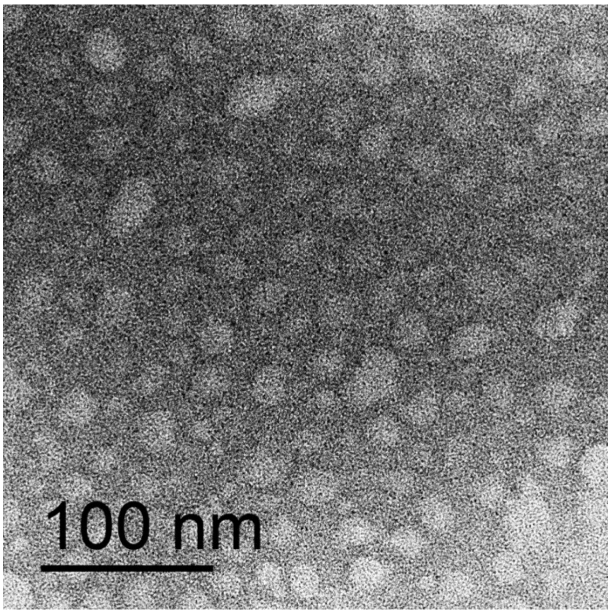

Fig. S8. TEM images of C-6F/DNA complexes at a mass ratio of 6.0 in deionized water

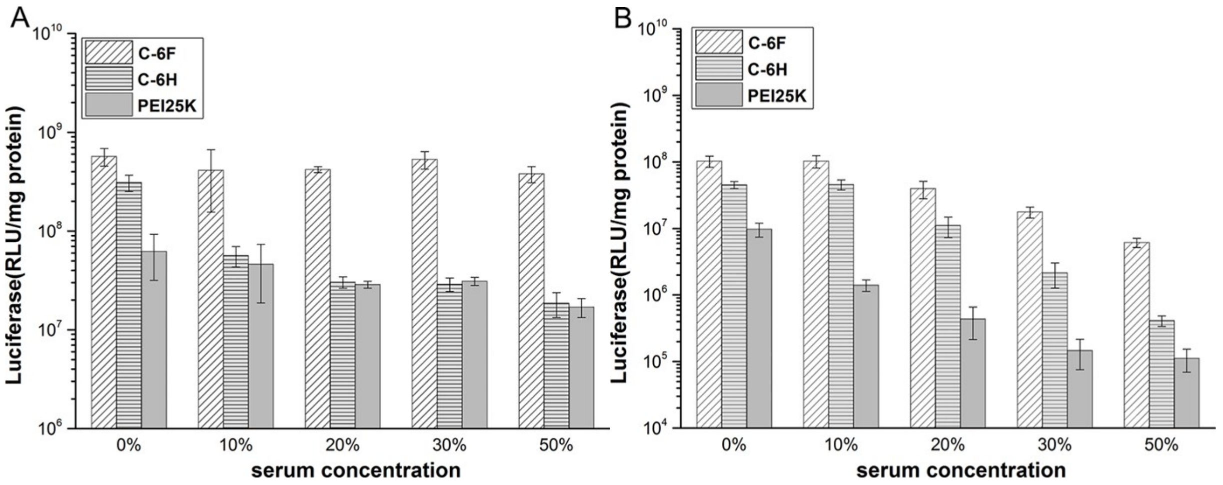

Fig. S9. Luciferase gene expression transfected by **C-6H** and **C-6F** derived complexes (w/w = 6) under various serum concentrations in comparison with 25 kDa PEI (w/w = 1.4). Transfections were carried out in 7702 (A) and HeLa (B) cells. Data shows the representative mean SD (n = 3).
